# Psychological Care for Children and Adolescents with Diabetes and Patient Outcomes: Results from the International Pediatric Registry SWEET

**DOI:** 10.1155/2023/8578231

**Published:** 2023-06-02

**Authors:** Agata Chobot, Alexander J. Eckert, Torben Biester, Sarah Corathers, Ana Covinhas, Carine de Beaufort, Zineb Imane, Jaehyun Kim, Anna Malatynska, Hossein Moravej, Santosh Pokhrel, Timothy Skinner, SWEET Study Group

**Affiliations:** ^1^Institute of Medical Sciences, University of Opole, Department of Pediatrics, Opole 45-418, Poland; ^2^University Clinical Hospital in Opole, Department of Pediatrics, Opole 45-418, Poland; ^3^Institute of Epidemiology and Medical Biometry, ZIBMT, Ulm University, Ulm 89081, Germany; ^4^German Center for Diabetes Research (DZD), Munich-Neuherberg 85764, Germany; ^5^AUF DER BULT, Diabetes Center for Children and Adolescents, Hannover 30173, Germany; ^6^Cincinnati Children's Hospital Medical Center, University of Cincinnati College of Medicine, Division of Endocrinology, Cincinnati, OH 45229, USA; ^7^APDP, Diabetes Portugal, Lisbon 1250-189, Portugal; ^8^Pediatric Clinic/Centre Hospitalier de Luxembourg, Department of Pediatric Diabetes and Endocrinology, Faculty of Technology, Science and Medicine, University of Luxembourg, Esch Belval, Luxembourg 1210, Luxembourg; ^9^UZ Brussels, Department of Pediatric Endocrinology, Brussels 1090, Belgium; ^10^Children's Hospital of Rabat, UM5S, Rabat, BP 6527, Morocco; ^11^Seoul National University Bundang Hospital, Seoul National University College of Medicine, Department of Pediatrics, Seongnam 13620, Republic of Korea; ^12^Neonatal Research Center, Shiraz University of Medical Sciences, Shiraz, Iran; ^13^Siddhartha Children and Women Hospital, Department of Pediatrics, Butwal 32907, Nepal; ^14^Institute of Psychology, University of Copenhagen, Copenhagen 1353, Denmark; ^15^Department of Psychology, La Trobe University, Bendigo, VIC 3086, Australia; ^16^Australian Centre for Behavioural Research in Diabetes, Melbourne, VIC 3051, Australia; ^17^SWEET e.V. Coordination Center, Diabetes Center for Children and Adolescents Kinder- und Jugendkrankenhaus Auf Der Bult, Hannover, Germany

## Abstract

**Background:**

Easy accessibility of psychosocial care is recommended for children and adolescents with type 1 diabetes (T1D) and their families.

**Objective:**

The study aimed to evaluate the availability of psychological care and its associations with glycemic control in centers from the multinational SWEET (Better control in Pediatric and Adolescent diabeteS: Working to crEate CEnTers of Reference) registry.

**Subjects:**

Centers participating in SWEET (*n* = 112) were invited to complete a structured online survey, designed for the study, regarding their psychology service.

**Methods:**

Linear/logistic regression models adjusted for several confounders were used to determine the patient's HbA1c (mmol/mol) and odds ratios (ORs) for diabetic ketoacidosis (DKA) and severe hypoglycemia (SH) related to survey responses.

**Results:**

76 (68%) centers with relevant data in the SWEET database responded to the survey. Psychological services were provided in 89% of the centers. The availability of psychological service in centers was associated with a slightly lower HbA1c of the patients (72 (62–82) vs. 67 (57–78) mmol/mol, *p* = 0.004) and significantly lower odds for DKA (1.8 (1.1–2.9), *p* = 0.027).

**Conclusions:**

Most centers from the SWEET registry offered some form of structured psychological care, consistent with the recommendations of easy access to psychosocial care for children and adolescents with T1D and their families. The main benefit of this psychological care appears to be in the incidence of DKA between centers. The study data also continues to emphasize the importance of treatment targets in shaping the outcomes of pediatric diabetes care. These findings should inform health-service planners and the diabetes community of the importance of mental healthcare in multidisciplinary diabetes teams.

## 1. Introduction

The International Society for Pediatric and Adolescent Diabetes (ISPAD) Clinical Practice Consensus Guidelines (CPCG) notes that “being diagnosed with diabetes in childhood or adolescence can interfere with the normative developmental changes and interact with psychological and social factors in youth and their families. Integrated and collaborative care is, therefore, necessary” [1]. Therefore, the ISPAD guidelines recommend easy access to psychosocial care for children and adolescents with type 1 diabetes (T1D) and their families [1]. Similar recommendations are also proposed by other international and national diabetes societies [2, 3]. These guidelines are based on the scientific evidence which demonstrates that diabetes impacts nearly all aspects of an individual's life [4]. Furthermore, it is shown that blood glucose values are not only dependent on the administered amounts of insulin, however, they are also influenced by numerous psychosocial factors [5–7].

The effectiveness of psychological interventions for people with diabetes is established with primary research and meta-analyses reporting benefits resulting from providing individual patients with psychological care or with specific interventions [8–11]. To our knowledge, there is little or no data showing whether the availability of psychological care in the individual diabetes center, is associated with the treatment outcomes and/or overall wellbeing of the center's patients.

Significant and sometimes substantial differences between pediatric diabetes centers have been shown in the past [12, 13]. The Hvidoere Study Group confirmed that gender, age, and family support impacted on the individual variability of hemoglobin A1c (HbA1c) [14–16]. They then demonstrated a significant association between the HbA1c treatment targets reported by the staff and the centers' HbA1c results at the patient level [14–16]. The latter result is replicated in two other studies [17, 18]. The Hvidoere Study Group also reported that another center-related factor associated with glycemic control was an effective collaboration of the multidisciplinary diabetes team members [15].

Although according to the ISPAD CPCG, children, adolescents, and young people with T1D and their families should have an easy and individualized access to psychological care, there is little published data to establish whether the availability of psychological services in pediatric diabetes centers is associated with patient outcomes. De Witt and colleagues [19] reported on a 2011 survey of ISPAD members in relation to their provision of psychological support. They reported that psychological care with an integrated mental health specialist occurred is less than half of the responding services, with larger centers more likely to provide integrated psychological services. Therefore, we decided to explore this question by using the data available from the multinational SWEET (Better control in Pediatric and Adolescent diabeteS: Working to crEate CEnTers of Reference) network and surveying them about their provision of psychological services [20]. Centers participating in SWEET, certified as centers of reference and collaborative or associated centers are required to comply to with a clear criteria including following the ISPAD CPCG. Centers upload their data regularly, twice yearly, into a database. In addition to analyzing the availability of psychological care in SWEET centers, this study attempted to assess if access or different psychological service features/elements were associated with glycemic control.

## 2. Methods

All centers who participated in the SWEET database in 2020 (*n* = 112; data from the 2019 treatment year) were invited to complete a structured online 17-item survey (Google Forms, Google LLC, California, United States) regarding their psychology service. The survey itself and the study flowchart are available online (Supplement and Supplementary Figure 1). Statistical analyzes included only centers with data in the SWEET database that provided answers to the questionnaire. Data were extracted from the SWEET database using the following criteria: 2019 treatment year and patients with T1D aged ≤18 years. The following data were aggregated for each patient (median for continuous variables and maximum for binary variables): age, biological sex, age at diagnosis, body mass index standard deviation score calculated using the World Health Organization reference values (BMI SDS), daily insulin dose (U/kg), HbA1c (mmol/mol) and (%), use of insulin pump (CSII), use of continuous glucose monitoring (CGM; includes both, real-time and intermittently scanned CGM), history of diabetes ketoacidosis (DKA; DKA at diabetes onset was not taken into account), and severe hypoglycemia (SH) as well as the number of self-measurements of blood glucose per day (SMBG). DKA and SH were presented as binary variables, and patients with 1 or more DKA or SH episode within the observation period was considered as patient with DKA or SH, respectively. Patients with missing data on pump use were excluded, while missing data on sensor use were considered as no sensor use. For all the other variables, missing data were only excluded from all analyses requiring the respective information. Questionnaire answers were grouped (detailed data available in the online Supplementary Data 1) and cross-tables were created.

The centers with available/presence of any psychological care (referred to further as PsyC centers) offered by a trained mental health specialist (MHS), either social worker, psychologist, or psychiatrist were grouped according to their compliance to the following four additional features of psychological care organization recommended by ISPAD CPCG: (a) having a psychologist and a psychiatrist and/or social worker in the MDT (multidisciplinary diabetes team), (b) offering psychological care at diabetes diagnosis, (c) annual consultation with a MHS and additional consultations as needed, and (d) using standardized psychological screening tools.

### 2.1. Statistical Analysis

All statistical analyses were generated using SAS (Statistical Analysis Software, SAS Institute Inc., Cary, NC, USA) Version 9.4, Built M7, on a Windows Server 2016 mainframe. Descriptive statistics were performed for all patients and for centers in the treatment year 2019. The results are shown as median with quartiles for continuous variables and as proportions for binary variables.

To measure associations with HbA1c (mmol/mol) and odds ratios (ORs) for DKA and SH related to survey responses, linear/logistic regression models were used. To take regional differences into account, a random intercept for regions with an unstructured variance-covariance matrix and an optimization technique of Newton−Raphson with ridging was implemented. Regions were defined as follows: Asia and Middle East + Africa, North America, South America, and Australia + New Zealand. All regression models were implemented for aggregated data of each patient in 2019 and adjusted for age (categorized: <10 years, 10 to <14 years, and ≥14 years), gender, age at T1D onset (categorized: <6 years, 6 to <10 years, and ≥10 years), pump use (yes/no), number of SMBG (categorized: ≤4, >4, and CGM), center size (≤500 patients, >500 to ≤1000 patients, and >1000 patients), HbA1c target (categorized: ≤7%, >7% or ≤53 mmol/mol, and >53 mmol/mol), and completeness of documentation defined as data on ≥50% of patients available (yes/no). To adjust for multiple comparisons the Tukey−Kramer method was used.

## 3. Results

Responses were received from 76 (68%) centers in the SWEET database (uploaded data for a total of 27,819 subjects) with 52% male, 12.9 (IQR 9.7; 15.5) years old, age at T1D onset 7.3 (4.1; 10.5), BMI SDS 0.53 (−0.17; 1.25), mean HbA1c 62 (53; 74) mmol/mol or 7.8 (7.0; 8.9) %, and daily insulin requirement 0.8 (0.63; 0.98) U/kg. In the centers that responded to the survey, 45% of patients used CSII and 46% were current CGM users. In terms of acute complications, 2.4% of patients has experienced at least one DKA and 1.3% at least one SH episode in the analyzed period (1 year). The characteristics of patients from these centers are comparable to that of all T1D pediatric patients from the SWEET registry, and a detailed comparison is provided in the online Supplementary Table 1.

### 3.1. Availability and Characteristics of Psychological Care

Psychological service was offered in 89% of the centers that responded to our survey. The characteristics of these centers and those without structured psychological services are shown in Table 1.

**Figure 1:**
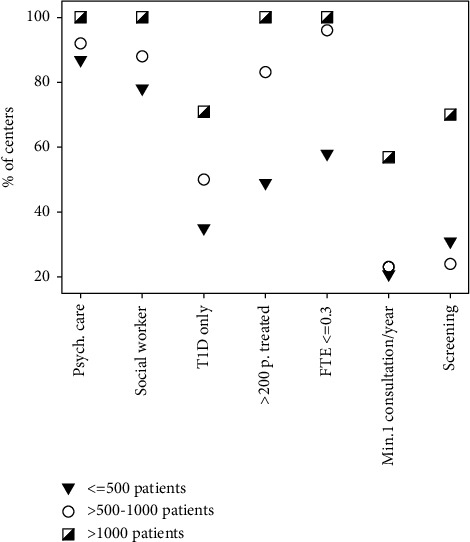
Questionnaire data by center size. Shown are the percentage of centers with ≤500 (black triangle), >500–1000 (white circle), and >1000 patients (grey square) providing the following outcomes. PsyC = psychological care was offered, SW = social worker in the healthcare team, T1D only = mental health specialist only took care of T1D patients, >200 *p* = mental health specialist took care of >200 patients, FTE ≤0.3 = mental health specialists-free time equivalent ≤0.3/100 patients, consult = at least on consultation at the mental health specialist per year, and screen = psychological screening tool used.

The analysis revealed differences in various aspects of psychological services depending on the center size. These are shown in Figure 1. The lack of psychological support and consultations was more frequent in small than in medium-sized centers and was not reported by any of the big centers. A social worker as part of the MDT was present in all big and majority of small and medium centers. The larger the PsyC center the more frequently the MHS was providing care exclusively for patients with diabetes. In large and medium centers, more often one MHS was responsible for more than 200 patients and families, whereas in small centers more full-time equivalents (FTE) of MHS per 100 patients were observed. Psychological screening tools are used in a majority of big centers and approximately a quarter of small and medium centers.

In 75% of the PsyC centers, psychological services were fully covered and in the remaining quarter the patients needed to contribute financially, at least partially for the psychological consultation.

The documentation of psychological consultations was integrated into the patients' medical records in 79% of PsyC centers. The majority of PsyC centers offered psychological care/support at T1D onset, mostly offering more than one consultation (31%) or MHS contact adjusted to the patient's and the family's needs (39%). Only a few (6%) reported having psychological services available at T1D's first appearance.

Practices regarding referral to a MHS differed between centers, and 40% of the centers psychological consultations took place at the request of the patient or their caregivers, and in 35% only on the request of the physician. In 25% of the clinics, the patients had at least one structured psychological consultation a year, with additional appointments scheduled at patients, physicians, or at other MDT member's request. The latter approach was frequently reported by small and medium centers and predominantly in big centers (Figure 1). Ongoing psychological services were available in most PsyC centers (78%), while the remainder offered only a one-off contact with a MHS. One-third of the PsyC centers used the structured psychological questionnaire tools to screen for mental health issues.

### 3.2. Availability and Features of Psychological Care in Centers in Terms of Patient Outcomes

Linear regression models showed that the availability of psychological services in centers was associated with a slightly lower HbA1c of the patients (68 vs. 72 mmol/mol, *p*=0.004). Having no access to psychological care was associated with higher odds for DKA (OR with 95% CI: 1.8 (1.1–2.9), *p*=0.027), but was not related to the rate of SH. Psychological care availability was associated with a lower BMI SDS (0.53 vs. 0.69, *p*=0.018). Patients in PsyC centers were more likely to use CGM (OR = 2.3 (1.9–2.9), *p*=0.001) and perform SMBG >4 times per day (2.0 (1.5–2.8), *p*=0.007).

Subsequently, we analyzed the associations of different features of psychological care on the patient's outcomes: HbA1c, DKA frequency, SH rate, and BMI SDS. Significantly lower HbA1c values were observed for the following aspects of psychological care: MHS working only with patients with diabetes, documentation from psychological consultations added to the patient's medical record, available ongoing psychological care, available contact with an MHS at T1D onset, and no additional cost for psychological care (detailed results are shown in Table 2).

Regarding acute and severe diabetes complications we found that lower OR for DKA was significantly associated with no patient financial contribution to the costs of psychological consultations (0.8 (0.6–1.0), *p*=0.049). No association of the rate of DKA was found with any of the following other variables describing availability and features of psychological care: who was the recipient of psychological consultations (exclusively patients with diabetes or also those with other conditions), the number of MHSs in the center, incorporating psychological consultations to the patients records, use of screening tools, type of referral for psychological consultations (any type compared to having a psychological consultation at least once per year with additional consultations upon referral by the patient or MDT members), presence or no psychological care at T1D diagnosis, and availability of ongoing psychological care.

Regression models revealed interesting associations of psychological care availability and its features with BMI SDS, as shown in online Supplementary Table 2 and Data 2. There were also differences in sensor use depending on the structure of psychological services (online Supplementary Data 3).

### 3.3. ISPAD Recommendations regarding Psychological Care

Only 4% of PsyC met all 4 recommendations related to access to a mental health specialist as a part of psychological care, as recommended by ISPAD CPCG. A further 15% met 3 recommendations and 34% met 2 recommendations. In 2 out of the 3 centers with full ISPAD CPCG compliance, the patients need to contribute financially at least partially to the psychological consultation. The number of MHSs/100 patients as well as MHS FTE/100 patients did not differ between centers and were dependent on the number of care recommendations they met.

Regression models showed that adherence to ISPAD CPCG (categorized: available psychological care+ up to any 2 additional features recommended by ISPAD as described in methods and psychological care+ any 3 or all 4 of additional features, compared to no psychological care) was associated with lower HbA1c and BMI SDS (Table 3 and online Supplementary Table 2 and Data 2). Increasing compliance with the guidelines was associated with decreasing OR for DKA and SH rates (Table 3).

### 3.4. HbA1c Target

The SWEET centers reported HbA1c targets ranging from 6.5% (48 mmol/mol) to 8.5% (69 mmol/mol), with <7% (53 mmol/mol) being the most common one. A lower HbA1c target (≤6.5% or 48 mmol/mol) is more frequently applied in smaller centers, and big centers do not use it at all and more frequently set higher HbA1c targets. Some centers reported using age-related targets for their patients, despite standard ISPAD guidelines.

Although among all HbA1c target groups, there were PsyC centers as well as those with no psychological services available, those with the lowest HbA1c target (≤6.5%, 48 mmol/mol) universally offered psychological care to their patients with MHS documentation to the medical record, and fully covered consultations costs (no patient contribution). However, none of the centers with target HbA1c ≤6.5% (48 mmol/mol) reported to comply with all ISPAD CGPG recommendations regarding the recommended features of psychological care.

Regression analysis showed that HbA1c target was associated with the HbA1c outcome of the center's patients with the lowest outcomes achieved by patients from centers that use the lowest HbA1c target (61, 69, and 66 mmol/mol, respectively, for the targets 6.5%/48 mmol/mol, 7 to <7.5%/53–57 mmol/mol, and ≥7.5%/≥58 mmol/mol, for all *p* < 0.001). There were also higher OR for DKA associated with higher HbA1c targets (7 to <7.5% vs. 6.5% OR = 2.0 (1.2–3.), *p* = 0.014 and ≥7.5% OR = 1.8 (1.0–3.1), *p*=0.039). The lowest target was associated with a lower OR for SH when compared to the highest target (6.5% vs. ≥7.5, OR = 0.51 (0.25–0.88), *p*=0.024). BMI SDS was not significantly associated with the HbA1c target.

## 4. Discussion

The most significant finding of this study was that access to psychological service in pediatric diabetes centers was associated with substantially lower rates of DKA. Whilst there was also a statistically significant association with HbA1c, this was not clinically meaningful. In addition, this study reaffirms previous research demonstrating an association between pediatric centers treatment targets and HbA1c. Although this latter result is not novel, it extends the previous research of the Hvidoere Childhood Diabetes Study group, to a wider sample of diabetes centers.

This study is the first to demonstrate an association between the availability of psychological care in a center and the outcomes of care for children and adolescents. There are clear mechanisms by which we would anticipate that having ready access to psychological care would result in reduced DKA. The literature on recurrent DKA is consistent in identifying that this is commonly associated with missed or omitted insulin injections and concurrent mental health issues [21–23]. Early referral, easy access, and affordable psychological care would enable the diabetes service to engage early with individuals at high risk for DKA and or pick up mental health problems early. Thus, with an early initiation of psychological intervention for these individuals, it is likely to prevent individuals from progressing to the point of DKA. Combined with the discrete change in HbA1c it may suggest that early psychological intervention allows to prevent the deterioration of glycemic control. MHSs, as members of the MDT may also help in retaining the motivation of patients. They may also help other MDT members to better adapt the treatment to the needs of specific patients and individualization of diabetes care was already described to be positively associated with glycemic control [15]. Yet the exact mechanisms need to be investigated further and these were not the aim of this study.

The data reported here provide an important initial evidence supporting the current recommendations regarding the availability of psychological services. Whilst there are many studies demonstrating the effectiveness of specific psychosocial interventions [24, 25], these trials rarely have samples representative of the general clinic population [26–29]. This study reports on the impact of the availability of psychological care for children with T1D in clinical practice. Thus, these data can be used to extrapolate the benefits of integrating psychological services into the pediatric diabetes care, and advocating for coverage of costs to remove the potential financial burden on families.

The findings of lower HbA1c targets are associated with a better glycemic control of the patients [14, 15, 17, 18]. The analysis of treatment targets and psychological care availability leads to a second point that is critical to note: having access to psychological care and meeting the ISPAD guidelines does not mean taking “a soft approach” and pursuing less demanding treatment targets for children and adolescents with diabetes. Centers with easy access to psychological care do not have less demanding treatment targets and these centers have individuals with lower HbA1c levels. Thus, psychological care is arguably enabling people to succeed, and it is supporting wellbeing as well as glycemic outcomes. Lastly, this study replicates the finding that centers that strive to achieve lower HbA1c targets are achieving better outcomes with their patient population. Clearly, there is a need to understand why some centers do not aim for lower HbA1c targets, and how do we support the adoption of current standards of care into widespread clinical practice.

The features of psychological care that are related to a better access of the patient to a MHS, such as available ongoing care, more than one MHS working in the center, as well as sharing the conclusions from psychological consultations with the whole MDT (adding them to the patients' documentation), were associated with better outcomes. It may suggest that psychological interventions when available may be introduced earlier, that is, before the patient's metabolic control deteriorates. It can be also hypothesized that due to the information from the MHS, other MDT members have a more holistic picture of the patient's life with diabetes and therefore adapt the training and communication to the different degrees of motivation and acceptance of diabetes. Further studies would be needed to provide evidence for these concepts.

The majority of SWEET centers offer some psychological care to their patients, with varying degrees of implementation of all recommendations from ISPAD CPCG regarding psychological care. It is not possible to compare these results with the previous survey of ISPAD members, as the guidelines and questions have changed and the sampling frame is substantially different. However, they do not suggest that there has been a substantial uptake of psychological services in pediatric diabetes centers, with only 4% of centers meeting the 4 guideline statements about access to a mental health specialist. There were significant differences in the organization of these services between centers, and one of the factors was the center size; and larger centers offer more psychological care. This replicates the previous survey of the ISPAD membership showing that larger centers were more likely to have integrated psychological services [19]. If psychological support was organized more consistently the OR for DKA and SH would be lower, but the difference in HbA1c and BMI would not be clinically relevant.

This is the first study to assess the overall association of psychological care availability on metabolic outcomes of pediatric patients with T1D. The results are based on the analysis of a large worldwide database from centers that participate in a twice-annual benchmarking and share the same goals for diabetes care. In addition, the response rate to the questionnaire among SWEET centers was high and therefore the studied group of centers can be considered representative. A limitation of this study is the discrepancy in the number of patients from centers with and without available psychological care, but regression models were adjusted for center size. Further limitations that need to be acknowledged include the following: lack of information on the percentage of patients receiving psychological care in single centers and its nature (ongoing care, support, and single consultation), and potential other factors related to the MDT composition. We acknowledge that other factors, for example, related to the approach to the patient or means of provided care could influence patient outcomes. Although the results were adjusted for multiple confounding factors, one cannot exclude that centers which implement psychosocial guidelines, have a different approach to diabetes care. Future studies should be designed to investigate the mechanism by which the psychological services impact patient outcomes.

## 5. Conclusions

In summary, most centers from the SWEET registry offer psychological care consistent with CPCG recommending easy access to psychosocial care for children and adolescents with T1D and their families. The data reported here suggests that having psychologists as part of the multidisciplinary team is associated with lower rates of life-threatening and compromising events such as DKA, and may be a funding priority for centers without access to such services.

## Figures and Tables

**Table 1:** Characteristics of centers offering/not offering psychological care.

	Centers offering psychological care 68 (89%) centers 26009 (95%) patients	Centers with no structured psychological services 8 (11%) centers 1296 (5%) patients
*Region of center N (%)*:		
Europe	41 (60.3)	2 (25.0)
South America	4 (5.9)	0 (0.0)
North America	3 (4.4)	1 (12.5)
Australia	5 (7.4)	1 (12.5)
Asia and Middle East + Africa	15 (22.0)	4 (50.0)
At least 50% of documented patients in SWEETBASE *N* (%)	43 (63.2)	3 (37.5)
*Center size N (%)*:		
≤500 patients	39 (57.4)	6 (75.0)
500–1000 patients	22 (32.4)	2 (25.0)
>1000 patients	7 (10.2)	0 (0.0)
*HbA1c target N (%)*:		
6.5% or 48 mmol/mol	10 (14.7)	0 (0.0)
7–7.5% or 53–58 mmol/mol	42 (61.8)	3 (37.5)
>7.5% or >58 mmol/mol	16 (23.5)	5 (62.5)
National guidelines recommend psychological service *N* (%)	63 (92.7)	4 (50.0)
National guidelines recommend social worker *N* (%)	46 (67.7)	3 (37.5)

For each box in the column, 100% means centers from all regions that, respectively, do or do not, provide psychological care.

**Table 2:** Results of regression models for chosen features of psychological care with significant differences in HbA1c (mean (95% confidence interval) (mmol/mol)) as outcome adjusted for gender, age at onset (cat.), age (cat.), pump, sensor, % of documented patients, HbA1c target, and center size (nonsignificant results are not shown). Hierarchical models with the region as a random effect.

Features of psychological care	HbA1c (mmol/mol) with 95% CI	*P* value
MHS working only with patients with diabetes vs. with diabetes and patients not having diabetes	67 [58–76] vs. 68 [59–78]	0.007
Adding the documentation from psychological consultations to the medical record of the patient: yes vs. no	67 [58–76] vs. 68 [59–78]	0.003
More than one MHS working in the center: yes vs. no	66 [57–76] vs. 68 [59–77]	0.008
Ongoing psychological care available vs. only single sessions	67 [57–80] vs. 68 [58–79]	0.036
Financing of the psychological care: fully covered vs. patient needs to contribute to the costs	65 [59–71] vs. 73 [67–79]	<0.001
Use of psychological screening tools: yes vs. no	68 [58–78] vs. 67 [57–77]	0.020

MHS: mental health specialist.

**Table 3:** Regression model analysis of associations between compliance to ISPAD Clinical Practice Consensus Guidelines (CPCG) and outcomes of pediatric patients with type 1 diabetes in SWEET centers. ISPAD CPCG recommends access to psychological care and to (a) have a psychologist and psychiatrist and/or social worker in the MDT, (b) offer psychological care at diabetes diagnosis, (c) provide patients with at least one consultation with an MHS annually and additional consultations as needed, and (d) use psychological screening tools.

	Psychological care available +3 to 4 additional features suggested by CPCG	Psychological care available+ up to 2 additional features suggested by CPCG	No psychological care
HbA1c (mmol/mol)	68 [61–76], *p*=0.002	67 [60–75], *p* < 0.001	72 [64–80]
BMI SDS	0.54 [0.22-0.86], *p*=0.016	0.53 [0.21-0.85], *p*=0.007	0.69 [0.36–1.02]
DKA	0.4 [0.2-0.7], *p*=0.007	0.6 [0.4-0.9], *p*=0.027	Reference
SH	0.4 [0.2-1.0], *p*=0.045	0.9 [0.4-1.8], *p* > 0.05	Reference

Results are presented as mean or odds ratio (OR) with 95% confidence interval (95% CI) with reference to “no psychological care.” BMI SDS: body mass index standard deviation score, DKA: diabetic ketoacidosis, and SH: severe hypoglycemia.

## Data Availability

The data that support the findings of the study are available from the corresponding author upon request.

## Supplementary Materials

Supplementary Materials Supplementary Figure 1: flowchart for selection of the study population from the SWEET registry. Supplementary Data 1: grouping of the questionnaire answers. Supplementary Table 1: characteristics of patients with type 1 diabetes aged <18 years from all SWEET centers in the 2020 database and patients from canters that responded to the survey. Supplementary Table 2 and Data 2: associations between availability and features of psychological care services in SWEET centers on BMI SDS. Supplementary Data 3: association between sensor use and features of psychological care services. Supplement: the survey. Appendix: a full list of contributing centers for the SWEET study group.
