# Supplementary material for: Psychological Care for Children and Adolescents with Diabetes and Patient Outcomes: Results from the International Pediatric Registry SWEET
Source: Pediatr Diabetes. 2023 Jun 2;2023:8578231. doi: 10.1155/2023/8578231 (PMC12017242; doi:10.1155/2023/8578231)
Supplement: Supplementary Materials — Supplementary Figure 1: flowchart for selection of the study population from the SWEET registry. Supplementary Data 1: grouping of the questionnaire answers. Supplementary Table 1: characteristics of patients with type 1 diabetes aged <18 years from all SWEET centers in the 2020 database and patients from canters that responded to the survey. Supplementary Table 2 and Data 2: associations between availability and features of psychological care services in SWEET centers on BMI SDS. Supplementary Data 3: association between sensor use and features of psychological care services. Supplement: the survey. Appendix: a full list of contributing centers for the SWEET study group. [file 8578231.f1.zip › Appendix - List of contributing centers.docx]

List of contributing centers:

Argentina, Buenos Aires: Hospital Juan P. Garrahan; Australia, Newcastle: John Hunter Childrens Hospital; Australia, Perth: Princess Margaret Hospital for Children; Australia, Queensland: South East Queensland Private Practice Group; Austria, Vienna: Universitätskinderklinik Wien; Belgium, Leuven: University Hospital Leuven (UZ Leuven); Brasil, Curitiba: Centro de Diabetes Curitiba; Bulgaria, Sofia: University Paediatric Hospital; Canada, Calgary: Alberta Health Services; Canada, Markham: Markham Stouffville Hospital; Chile, San Felipe: Hospital San Camilo; Costa Rica, San Jose: National Childrens Hospital, Hospital CIMA; Croatia, Zagreb: University Clinical Hospital Center Sestre milosrdnice; Croatia, Zagreb: University Hospital Zagreb; Czech Republic, Prague: University Hospital Motol Prague; Denmark, Aarhus: University of Aarhus; Denmark, Herlev: Herlev University Hospital; Ecuador, Quito: Fundación Diabetes Juvenil Ecuador; Egypt , Cairo: Ain Shams University; England, London: Barts and the London NHS Trust; England, Mansfield: Sherwood Forest Hospital; France, Angers: University Hospital Angers; France, Bordaux: Centre Hospitalier Universitaire de Bordeaux; Germany, Hannover: Kinderkrankenhaus Auf der Bult; Greece, Athens: Athens University; Greece, Athens: P&A Kyriakou Childrens Hospital; Greece, Thessaloniki: AHEPA University Hospital, 2nd Department of Paediatrics, Aristotle University of Thessaloniki; Greece, Thessaloniki: Hippokration Hospital of Thessaloniki; Hungary, Budapest: Semmelweis University; India, Ahmedabad Bareja: Rudraksha Insitute of Medical Sciences; India, Ahmedabad: Diacare Clinic; India, Ahemedabad: Swasthya Diabetes Care; India, Belgaum: KLE Universitys Jawaharlal Nehru Medical College; India, Chennai: MV Diabetes center; India, Coimbatore: PSG institute of medical sciences; India, Kota Rajasthan: Ramchandani Diabetes Care and Research Centre; India, Trivandrum: Jothydevs Diabetes And Research Centre; Iran, Shiraz: Shiraz University of Medical Sciences; Ireland, Cork: Cork University Hospital; Israel, Petah: Schneider Childrens Medical Center of Israel,Endocrinology; Italy, Ancona: Salesi University Hospital; Italy, Florence: Meyer Children Hospital; Italy, Mailand: Ospedale San Raffaele; Italy, Rome: Bambino Gesu Childrens Hospital; Italy, Turin: Centro Diabetologia Pediatrica; Italy, Verona: Universita di Verona; Korea, Seongnam: Seoul National University Bundang Hospital; Kuwait, Kuwait City: Dasman Institute; Latvia, Riga: Children clinical University Hospital; Lithuania, Kaunas: Hospital of LUHS Kauno Klinikos; Luxembourg, Centre Hospitalier de Luxembourg; Mali, Bamako: NGO Santé Diabète / Hopital du Mali; Mauritius, Vacoas: T1Diams; Morocco, Rabat: Children’s Hospital – Unit Of Pediatric Diabetology; Nepal, Butwal: Siddharta Children & Women Hospital; New Zealand, Auckland: Auckland Starship Hospital; New Zealand, Christchurch: University of Otago and Canterbury District Health Board; Poland, Katowice: Medical University of Silesia; Poland, Opole: USK Opole; Poland, Rzeszow: University of Rzeszow, Pediatric Endocrinology and Diabetes; Poland, Warsaw: Medical University of Warsaw; Portugal, Porto: Centro Hospitalae S. Joao; Romania, Bucharest: Diabetes Nutrition and Metabolic Diseases Clinic DiabNutriMed; Romania, Bucharest: Elias University Emergency Hospital; Romania, Buzias: Clinical Center Cristian Serban; Serbia, Belgrade: Institute for Mother and Child Healthcare; Slovenia, Ljubljana: University Childrens Hospital; Spain, Barakaldo: Hospital Universitario Cruces; Spain, Barcelona: Hospital Sant Joan de Deu; Sweden, Gothenburg: The Queen Silvia Childrens Hospital; Sweden, Uddevalla: Uddevalla Childrens Hospital; Tanzania, Daressalam: Muhimbili National Hospital; Turkey, Duzce: University of Duzce, Department of Pediatric Endocrinology; USA, Cincinnati: Cincinnati Childrens; USA, Denver Colorado: Barbara Davis Center
