# Supplementary material for: Psychological Care for Children and Adolescents with Diabetes and Patient Outcomes: Results from the International Pediatric Registry SWEET
Source: Pediatr Diabetes. 2023 Jun 2;2023:8578231. doi: 10.1155/2023/8578231 (PMC12017242; doi:10.1155/2023/8578231)
Supplement: Supplementary Materials — Supplementary Figure 1: flowchart for selection of the study population from the SWEET registry. Supplementary Data 1: grouping of the questionnaire answers. Supplementary Table 1: characteristics of patients with type 1 diabetes aged <18 years from all SWEET centers in the 2020 database and patients from canters that responded to the survey. Supplementary Table 2 and Data 2: associations between availability and features of psychological care services in SWEET centers on BMI SDS. Supplementary Data 3: association between sensor use and features of psychological care services. Supplement: the survey. Appendix: a full list of contributing centers for the SWEET study group. [file 8578231.f1.zip › Supplement.pdf]

Psychological care in SWEET Centers & diabetological care during the COVID-19 pandemic in SWEET Centers questionnaire

You received the link to this questionnaire as a member of the SWEET community. Please provide information regarding psychological care and its structure in the SWEET Center You work in as well as some facts regarding diabetological care during the COVID-19 pandemic. Your involvement will allow to analyze and better undrestand these aspects of care offered in SWEET Centers.

*Wymagane

Part 1: Psychological care in SWEET Centers

1. 1. Center: *

Zaznacz tylko jedną odpowiedź.

- Algeria, Algiers
- Argentina, Buenos Aires, 10350
- Australia, Gold Coast, 10375
- Australia, Perth, 10379
- Australia, Brisbane-Wolloongabba, 10396
- Australia, Geelong, 10411
- Australia, Newcastle, 10424
- Australia, Burnie
- Austria, Vienna, 10354
- Bangladesh, Dhaka, 10401
- Belgium, Leuven, 10335
- Bolivia, Cochabamba
- Brazil, Curitiba-Parana, 10357
- Bulgaria, Sofia, 10358
- Bulgaria, Varna, 10359
- Cameroon, Yaounde
- Canada, Sherbrooke, 10381
- Canada, Vancouver, 10393
- Canada, Markham, 10406
- Canada, Montreal
- Canada, Winnipeg
- Canada, Montreal
- Canada, Ottawa
- Canada, Halifax
- Canada, Edmonton
- Canada, Calgary, 10362
- Chile, San Felipe, 10398
- Costa Rica, San José, 10349
- Croatia, Zagreb, 10289
- Croatia, Zagreb, 10355
- Czech Republic, Prague, 10318

- Czech Republic, Prague, 10217
- Denmark, Herlev, 10292
- Denmark, Arhus, 10347
- Ecuador, Quito, 10403
- Ecuador, Cuenca, 10433
- Egypt, Cairo, 10378
- Egypt, Cairo, 10408
- France, Paris, 10225
- France, Bordeaux, 10377
- France, Angers, 10395
- France, Clermont-Ferrrand
- France, Tours
- France, Toulouse
- France, Le Kremlin Bicêtre
- Germany, Hannover, 10161
- Germany, Leverkusen, 0368
- Ghana, Kumasi
- Greece, Athens, 10223
- Greece, Athens, 10346
- Greece, Thessaloniki, 10419
- Greece, Thessaloniki, 10421
- Haiti, Port-au-Prince, 10427
- Haiti, Montrouis
- Hungary, Budapest, 10214
- India, Ahmedabad, 10344
- India, Belgaum, 10363
- India, Ahmedabad, 10369
- India, Ahmedabad, 10386
- India, Jaipur, 10387
- India, Kota, 10388
- India, Nagpur, 10389
- India, Aurangabad, 10390
- India, Coimbatore, 10394
- India, Kanpur, 10397
- India, Chennai, 10404
- India, Trivandrum, 10409
- India, Sangli Kolhapur, 10410
- India, Ahmedabad, 10420
- India, Mumbai, 10425
- India, Bareja Ahmedabad, 10432
- India, Visakhapatnam
- India, Valsad
- India, Jalandhar City
- India, Hyderabad
- India, Guntur
- India, Bangalore
- India, Mumbai
- India, Nashik
- India, Mumbai
- India, Delhi
- Iran, Shiraz, 10415
- Iraq, Duhok
- Ireland, Cork, 10343
- Ireland, Dublin, 10356
- Ireland, Limerick, 10431
- Israel, Petach Tikva, 10364
- Italy, Verona, 10218
- Italy, Ancona, 10341
- Italy, Florence, 10342
- Italy, Milan, 10399
- Italy, Rome, 10400
- Italy, Turin, 10405
- Japan, Osaka, 10384
- Kenya, Nairobi
- Kuwait, Kuwait City, 10413
- Latvia, Riga, 10352
- Lithuania, Kaunas, 10353
- Luxembourg, Luxembourg, 10222

- Maldives, Male, 10428
- Mali, Bamako, 10361
- Mauritius, Vacoas, 10370
- Morocco, Rabat, 10417
- Nepal, Butwal, 10423
- Nepal, Suameri
- New Zealand, Auckland, 10376
- New Zealand, Christchurch, 10429
- Nigeria, Abakaliki
- Norway, Haugesund, 10414
- Norway , Oslo
- Pakistan, Lahore, 10385
- Pakistan, Sialkot
- Pakistan, Karachi
- Poland, Warsaw, 10219
- Poland, Warsaw, 10348
- Poland, Katowice, 10380
- Poland, Opole, 10412
- Poland, Lodz
- Poland, Rzeszów, 10416
- Portugal, Lisbon, 10213
- Portugal, Coimbra, 10313
- Portugal, Lisbon, 10314
- Portugal, Porto, 10345
- Republic of the Congo, Pointe-Noire, 10422
- Romania, Bucharest, 10365
- Romania, Bucharest, 10430
- Romania, Cluj-Napoca
- Romania, Buzias, 10221
- Serbia, Belgrade, 10373
- Slovenia, Ljubljana, 10329
- South Korea, Seongnam, 10391
- Spain, Barcelona, 10351
- Spain, Barakaldo-Bilbao, 10407
- Spain, Barcelona
- Sweden, Gothenborg, 10216
- Sweden, Uddevalla, 10366
- Tanzania, Daressalam, 10402
- Thailand, Bangkok
- The Netherlands, Rotterdam, 10215
- Turkey, Izmir, 10322
- Turkey, Duzce, 10382
- United Kingdom, Mansfield, 10371
- United Kingdom, London, 10212
- United Kingdom, Leeds, 10360
- United Kingdom, Birmingham, 10392
- United Kingdom, London
- United Kingdom, Manchester
- USA, Denver, 10367
- USA, Stanford, 10383
- USA, Cincinnati, 10418
- USA, Boston, 10426
- USA, New York
- USA, Gainesville
- USA, Kansas City
- USA, Seattle
- Center is not listed

2. 2. What is the current number of patients with type 1 diabetes treated in the Center: *

3. 3. What is the target HbA1c in Your Center? *

Zaznacz tylko jedną odpowiedź.

- ≤6.5%
- ≤7%
- ≤7.5%
- ≤8.0%
- Inne: ______

4. 3a. If the HbA1c target in the Center is age-dependent please specify:

______

5. 4. Do Your local (i.e. national) guidelines recommend a psychologist to be a member of the multidisciplinary diabetes team? *

Zaznacz tylko jedną odpowiedź.

- Yes
- No

6. 5. Do Your local (i.e. national) guidelines recommend a social worker to be a member of the multidisciplinary diabetes team? *

Zaznacz tylko jedną odpowiedź.

- Yes
- No

7. 6. Does Your multidisciplinary diabetes team involve a social worker? *

Zaznacz tylko jedną odpowiedź.

- No
- Yes, but the social worker is available only on demand
- Yes, and the social worker is available on a regular basis

8. 7. Does the Center offer psychological care? *

Zaznacz tylko jedną odpowiedź.

- No, patients have no structured access to psychological care within the offer of the Center
- Yes - the Center has an agreement/contract with an external institution/ psychologist, where patients are referred to
- Yes – there is/are specialists providing psychological care (psychologist/s or psychiatrists or mental health nurse/s) employed in the Center
- Yes – there is/are specialists providing psychological care (psychologist/s or psychiatrists or mental health nurse/s) employed in the Center, and the Center has also an agreement/contract with an external institution/ psychologist, where patients are referred to

If the answer to the last question was "No" (the Center does not provide a structured psychological care) please tick "Not applicable" or put "0" in questions 8-16

9. 8. How many specialists provide psychological care for patients with type 1 diabetes in the Center? *

Zaznacz tylko jedną odpowiedź.

- 0
- 1
- 2
- 3
- 4
- 5
- 6
- 7
- 8
- 9
- 10
- more than 10

10. 9. The total full time equivalent of the specialists providing psychological care in the Center equals: *

11. 10. The specialist providing psychological care sees (in the Institution where the Centre is based) – SIGN ALL APPROPRIATE: *

Zaznacz wszystkie właściwe odpowiedzi.

- [ ] Not applicable (Center does not offer structured psychological care)
- [ ] Children and adolescents with diabetes
- [ ] Adults with diabetes
- [ ] Patients with conditions other than diabetes
- [ ] Hospitalized patients (in-patient care)
- [ ] Patients in the out-patient clinic (ambulatory care)

12. 11. Does the specialist who provides psychological care add his documentation to the medical history of patients (either hand written or electronic documentation)? *

Zaznacz tylko jedną odpowiedź.

- Yes
- No
- Not applicable (Center does not offer structured psychological care)

13. 12. What is the professional background of the specialist's /specialists' who provide(s) psychological care in the Center? (tick all appropriate) *

Zaznacz wszystkie właściwe odpowiedzi.

- [ ] Not applicable (Center does not offer structured psychological care)
- [ ] Psychology
- [ ] Psychotherapy
- [ ] Clinical Psychology
- [ ] Psychodietetics
- [ ] Mental Health Nurse
- [ ] Psychiatrist
- [ ] Health Psychologist
- [ ] Social Worker

Inne: [ ]

14. 13. At type 1 diabetes diagnosis the child/adult with diabetes and his family: *

Zaznacz tylko jedną odpowiedź.

- Not applicable (Center does not offer structured psychological care)
- Have no contact with a specialist providing psychological care
- Have a standardized contact with a specialist providing psychological care that includes more than 1 consultation
- Have contact with a specialist providing psychological care only on the idividual's demand
- Have a single contact with a specialist providing psychological care
- Have contact with a specialist providing psychological care adequately to the needs of the person with diabetes/family identified by the other members of the mutidisciplinary diabetes team (one or more frequent)

15. 14. Sign the most appropriate describing the psychological care in the Center: *

Zaznacz tylko jedną odpowiedź.

- Not applicable (Center does not offer structured psychological care)
- The patients are referred to a psychological consultation only by the physician (if the physician identifies that the patient requires a psychological consultation or the patient asks for it)
- The patients can contact and make an appointment with the center's specialist providing psychological care by themselves, but are also referred by the physician and/or other multidisciplinary team members
- All patients are consulted by the specialist providing psychological care at least once per year (as standard) and further visits are appointed depending on the patients' needs or if referred to by the physician

Psychological care in SWEET Centers & diabetological care during th... https://docs.google.com/forms/u/0/d/1cH7jHgNtTQ0NNr7ltc5vxZ6R...

16. 15. Is a screening tool (for example a structured questionnaire) used during the visits of the patients to screen for psychological problems and facilitate the decision whether the patient requires a psychological consultation? *

Zaznacz tylko jedną odpowiedź.

- Yes
- No
- Not applicable (Center does not offer structured psychological care)

17. 16. What is the type of psychological care offered in Your Center (tick all appropriate): *

Zaznacz wszystkie właściwe odpowiedzi.

- [ ] psycho-diagnostic assessment
- [ ] single-session psychological counselling
- [ ] psychotherapy
- [ ] other ongoing psychological care
- [ ] ongoing psychiatric care
- [ ] Not applicable (Center does not offer structured psychological care)

18. 17. How is the access to psychological care financed in Your Center? *

Zaznacz tylko jedną odpowiedź.

- The salary of the person providing the psychological service is paid for by the overall funding of the diabetes team service, the family make no contribution
- The salary of the person providing the psychological service is part paid by the overall funding of the diabetes service and the family make a contribution
- The salary of the person providing the psychological service is not included in the funding of the diabetes service and has to be paid for by the family
- Not applicable (Center does not offer structured psychological care)

Psychological care in SWEET Centers & diabetological care during th... https://docs.google.com/forms/u/0/d/1cH7jHgNtTQ0NNr7ltc5vxZ6R...

19. Please state any individual comments:

____

____

____

____

____

Part 2: Diabetological care during COVID-19 pandemic in SWEET centers:

20. 18. Did Your Center offer telemedicine consultations before the COVID-19 pandemic? *

Zaznacz tylko jedną odpowiedź.

- [ ] No
- [ ] Yes, but the majority were traditional, in-person consultations
- [ ] Yes, telemedicine consultations were equally frequent as in-person consultations
- [ ] Yes, the majority were telemedicine consultations

21. 19. How did the COVID-19 pandemic impact the type of ambulatory consultations offered in Your Center? *

Zaznacz tylko jedną odpowiedź.

- [ ] There was no change
- [ ] We only partially switched to telemedicine (phone calls, video calls, text messages, emails, other), in-person visits were only a bit less frequent
- [ ] We switched strongly towards telemedicine (phone calls, video calls, text messages, emails, other) – in-person visits only in exceptional cases

22. 20. How did the COVID-19 pandemic impact the type of in-patient care (hospitalizations) offered byYour center? *

Zaznacz tylko jedną odpowiedź.

- [ ] There was no change
- [ ] We continue/d acute admissions as well as regular (planned, control) admissions, but the number dropped
- [ ] We continue/d only acute admissions. Regular (planned, control) admissions are/were suspended.

23. 21. Did the Center offer additional psychological support for the patients because of the COVID-19 pandemic? *

Zaznacz tylko jedną odpowiedź.

- [ ] Yes
- [ ] No

24. 22. Do You plan to maintain telemedicine consultations in the future? *

Zaznacz tylko jedną odpowiedź.

- [ ] Yes
- [ ] No

25. 23. Is telemedicine reimbursed in Your country (or region, if reimbursement varies throughout the country)? *

Zaznacz tylko jedną odpowiedź.

- [ ] No
- [ ] Yes, a telemedicine visit is reimbursed less than an in-person consultation
- [ ] Yes, a telemedicine visit is reimbursed equally as an in-person consultation
- [ ] Yes, a telemedicine visit is reimbursed more than an in-person consultation

26. Individual comments:

Przejdź do sekcji 5 (Thank You!)

Thank You!

Thank You for participating and submitting Your answers!

SWEET

Ta treść nie została utworzona ani zatwierdzona przez Google.

Formularze Google
