# Supplementary material for: Psychological Care for Children and Adolescents with Diabetes and Patient Outcomes: Results from the International Pediatric Registry SWEET
Source: Pediatr Diabetes. 2023 Jun 2;2023:8578231. doi: 10.1155/2023/8578231 (PMC12017242; doi:10.1155/2023/8578231)
Supplement: Supplementary Materials — Supplementary Figure 1: flowchart for selection of the study population from the SWEET registry. Supplementary Data 1: grouping of the questionnaire answers. Supplementary Table 1: characteristics of patients with type 1 diabetes aged <18 years from all SWEET centers in the 2020 database and patients from canters that responded to the survey. Supplementary Table 2 and Data 2: associations between availability and features of psychological care services in SWEET centers on BMI SDS. Supplementary Data 3: association between sensor use and features of psychological care services. Supplement: the survey. Appendix: a full list of contributing centers for the SWEET study group. [file 8578231.f1.zip › Supplementary Data 1.docx]

*Supplementary Data 1. Grouping of the questionnaire answers.*

Number of patients with type 1 diabetes treated in center (center size): small (≤500 patients), medium (>500 to ≤1000 patients), and big (>1000 patients).

Center HbA1c target: 6.5%, 7%, 7.5% and above for descriptive statistics; as no big center had a target <6.5% for further statistical analysis only 2 groups were taken into account 7% (Centers with targets <6.5% and <7%) and target higher than 7%.

Multidisciplinary diabetes team involves a social worker: No, Yes (centers having a social worker on demand or on regular basis).

Number of psychological care specialists per 100 patients: none, up to 0.5 mental health specialist per 100 patients and more than 0.5 mental health specialists per 100 patients.

Amount of full time equivalents of psychological care specialists per 100 patients: none, 0.1 to 0.3 full time equivalents per 100 patients, more than 0.3 full time equivalent of mental health specialist per 100 patients.

Centers not providing psychological care were excluded from the grouping by answers regarding the structure of provided psychological care - below.

What kind of patients are consulted by the specialist providing psychological care: only patients with diabetes (children and/or adults, in- and out-patient), patients with diabetes as well as those with other conditions (any set of answers involving “patients with other conditions than diabetes”).

Type of specialist providing psychological care: only “psychologist” (includes: psychologist, health psychologist, psychodietetics, clinical psychologist, psychotherapist, mental health nurse), psychologist (defined as above) AND psychiatrist OR social worker.

Psychological care at diabetes diagnosis: no contact at diagnosis, contact and consultations adequately to the needs of the patient and family, any other type of psychological care offered at diagnosis (standardized contact with more than one consultation or contact on demand or single consultation.

How is the patient referred to the mental health specialist: only physician refers patients to psychologist / physician refers for psychological consultation but patient can contact psychologist also alone / at least annual visit and additional consultations as scheduled by the physician of patient alone; “not applicable” – the center does not provide structured psychological care.

Type of psychological care offered in center: ongoing psychological care (includes ongoing psychiatric care, other ongoing psychological care, psychotherapy), other type than ongoing psychological support (single session counselling or psycho-diagnostic assessment).

Financing of psychological care: psychological consultations covered fully or partially by family, the patient does not participate in the psychological care costs.
