# Supplementary material for: Psychological Care for Children and Adolescents with Diabetes and Patient Outcomes: Results from the International Pediatric Registry SWEET
Source: Pediatr Diabetes. 2023 Jun 2;2023:8578231. doi: 10.1155/2023/8578231 (PMC12017242; doi:10.1155/2023/8578231)
Supplement: Supplementary Materials — Supplementary Figure 1: flowchart for selection of the study population from the SWEET registry. Supplementary Data 1: grouping of the questionnaire answers. Supplementary Table 1: characteristics of patients with type 1 diabetes aged <18 years from all SWEET centers in the 2020 database and patients from canters that responded to the survey. Supplementary Table 2 and Data 2: associations between availability and features of psychological care services in SWEET centers on BMI SDS. Supplementary Data 3: association between sensor use and features of psychological care services. Supplement: the survey. Appendix: a full list of contributing centers for the SWEET study group. [file 8578231.f1.zip › Supplementary Data 3..docx]

*Supplementary Data 3. Association between sensor use and features of psychological care services.*

Sensor use was greater in centers where: psychological documentation is part of the medical records (OR=1.99 [1.71-2.31], p=0.0007), there is more than 1 MHS (OR=1.94 [1.73-2.19], p<0.001), and ongoing psychological care is available (OR=1.98 [1.76-2.23], p=0.0004). The sensor use was lower in centers where the referral to psychological consultations was initiated solely by the patient when compared to referrals either by the physician (OR=0.81 [0.73-0.91], p<0.001) or having a MHS consultation at least once annually (OR=0.71 [0.66-0.82], p<0.001). Sensor use was lower in centers with any type of psychological support at T1D diagnosis when compared to no contact at all (single contact OR=0.10 [0.08-0.14], more than one contact OR=0.17 [0.13-0.23], contact with MHS according to the patients’ needs OR=0.18 [0.13-0.23], p<0.001 for all) – even high technology application is not related to availability of psychological care at disease onset.

All logistic regression models for calculation of OR for sensor use were implemented for aggregated data of each patient in 2019 and adjusted for age (categorized: <10 years, 10 to <14 years, ≥14 years), gender, age at type 1 diabetes onset (categorized: <6 years, 6 to <10 years, and ≥10 years), pump use (yes/no), number of SMBG (categorized: ≤4, >4, CGM), center size (categorized: ≤500 patients, >500 patients), HbA1c target (categorized: ≤7%, >7% or ≤53mmol/mol, >53mmol/mol) and completeness of documentation defined as data on ≥50% of patients available (yes/no). To take regional differences into account, a random intercept for regions with Cholesky variance structure and an optimization technique of Newton-Raphson with ridging was implemented. Regions were defined as: Europe, Asia & Middle East + Africa, North America, South America, Australia + New Zealand. To adjust for multiple comparisons the Tukey-Kramer method was used.
