# Supplementary material for: Psychological Care for Children and Adolescents with Diabetes and Patient Outcomes: Results from the International Pediatric Registry SWEET
Source: Pediatr Diabetes. 2023 Jun 2;2023:8578231. doi: 10.1155/2023/8578231 (PMC12017242; doi:10.1155/2023/8578231)
Supplement: Supplementary Materials — Supplementary Figure 1: flowchart for selection of the study population from the SWEET registry. Supplementary Data 1: grouping of the questionnaire answers. Supplementary Table 1: characteristics of patients with type 1 diabetes aged <18 years from all SWEET centers in the 2020 database and patients from canters that responded to the survey. Supplementary Table 2 and Data 2: associations between availability and features of psychological care services in SWEET centers on BMI SDS. Supplementary Data 3: association between sensor use and features of psychological care services. Supplement: the survey. Appendix: a full list of contributing centers for the SWEET study group. [file 8578231.f1.zip › Supplementary Figure1.docx]

*Supplementary Figure 1. Flowchart for selection of the study population from the SWEET registry.*

All patients

in the SWEET database

112 centers, N=77,254

Other types of diabetes

N=7,222

Type 1 diabetes

112 centers, N=70,029

Age > 18 years

N=8,319

Age ≤18 years

112 centers, N=61,710

No SWEET data or information

on insulin pump use in 2019

8 centers, N=27,551

Treatment year 2019

104 centers, N=34,159

112 centers, N=

Did not respond to survey

28 centers, N=6,854

Responded to the survey

76 centers (68%), N=27,305

112 centers, N=
