# Supplementary material for: Psychological Care for Children and Adolescents with Diabetes and Patient Outcomes: Results from the International Pediatric Registry SWEET
Source: Pediatr Diabetes. 2023 Jun 2;2023:8578231. doi: 10.1155/2023/8578231 (PMC12017242; doi:10.1155/2023/8578231)
Supplement: Supplementary Materials — Supplementary Figure 1: flowchart for selection of the study population from the SWEET registry. Supplementary Data 1: grouping of the questionnaire answers. Supplementary Table 1: characteristics of patients with type 1 diabetes aged <18 years from all SWEET centers in the 2020 database and patients from canters that responded to the survey. Supplementary Table 2 and Data 2: associations between availability and features of psychological care services in SWEET centers on BMI SDS. Supplementary Data 3: association between sensor use and features of psychological care services. Supplement: the survey. Appendix: a full list of contributing centers for the SWEET study group. [file 8578231.f1.zip › Supplementary Table 1.docx]

*Supplementary Table 1. Characteristics of patients with type 1 diabetes aged < 18 years from all SWEET centers in the 2020 database and patients from canters that responded to the survey.*

|  | SWEET 2020 database – all patients fulfilling the inclusion data with no regard of respondence to the survey  104 centers; 34,159 pts | | | Centers that responded to the survey  76 centers (73%); 27,305 pts | | | Patients from centers that did not respond to the survey  28 centers (27%); 6854 pts | | |
| --- | --- | --- | --- | --- | --- | --- | --- | --- | --- |
|  | % or median | Q1 | Q3 | % or median | Q1 | Q3 | % or median | Q1 | Q3 |
| Gender M/F (%) | 52/48 |  |  | 52/48 |  |  | 52/48 |  |  |
| Pump use (%) | 45 |  |  | 45 |  |  | 44 |  |  |
| CGM use (%) | 47 |  |  | 46 |  |  | 31 |  |  |
| DKA (%) | 2.5 |  |  | 2.4 |  |  | 3.0 |  |  |
| Severe Hypoglycaemia (%) | 1.3 |  |  | 1.3 |  |  | 1.6 |  |  |
| Age (years) | 12.9 | 9.8 | 15.6 | 12.9 | 9.7 | 15.5 | 13.2 | 10.0 | 15.8 |
| Age at type 1 diabetes onset | 7.3 | 4.1 | 10.5 | 7.3 | 4.1 | 10.4 | 7.7 | 4.4 | 10.8 |
| BMI SDS | 0.54 | -0.17 | 1.27 | 0.53 | -0.17 | 1.25 | 0.60 | -0.17 | 1.35 |
| DIR (U/kg) | 0.80 | 0.63 | 0.99 | 0.80 | 0.63 | 0.98 | 0.84 | 0.65 | 1.06 |
| HbA1c (mmol/mol) | 62 | 53 | 74 | 62 | 53 | 74 | 63 | 55 | 74 |
| HbA1c (%) | 7.8 | 7.0 | 8.9 | 7.8 | 7.0 | 8.9 | 7.9 | 7.2 | 8.9 |
| Number of SMBG per day | 4 | 2 | 5.6 | 4 | 2 | 6 | 3 | 1 | 5 |

CGM – continuous glucose monitoring, DKA – diabetes ketoacidosis, T1D – type 1 diabetes, DIR – daily insulin requirement, SMBG – Self Monitoring of Blood Glucose, Q1, Q3 – first, third quartile.
