# Supplementary material for: Psychological Care for Children and Adolescents with Diabetes and Patient Outcomes: Results from the International Pediatric Registry SWEET
Source: Pediatr Diabetes. 2023 Jun 2;2023:8578231. doi: 10.1155/2023/8578231 (PMC12017242; doi:10.1155/2023/8578231)
Supplement: Supplementary Materials — Supplementary Figure 1: flowchart for selection of the study population from the SWEET registry. Supplementary Data 1: grouping of the questionnaire answers. Supplementary Table 1: characteristics of patients with type 1 diabetes aged <18 years from all SWEET centers in the 2020 database and patients from canters that responded to the survey. Supplementary Table 2 and Data 2: associations between availability and features of psychological care services in SWEET centers on BMI SDS. Supplementary Data 3: association between sensor use and features of psychological care services. Supplement: the survey. Appendix: a full list of contributing centers for the SWEET study group. [file 8578231.f1.zip › Supplementary Table 2. and Data 2. (1).docx]

*Supplementary Table 2. and Data 2. Associations between availability and features of psychological care services in SWEET centers on BMI SDS.*

| Features of psychological care |  |  | P |
| --- | --- | --- | --- |
| Adding the documentations from psychological consultations to the medical record of the patient – Yes vs No | 0.46 [0.01-0.91] | 0.54 [0.10-1.00] | 0.035 |
| At least one psychological consultation annually with additional on patient’s or MDT’s referral vs any other referral (less individualized) | 0.59 [0.25-0.93] | 0.50 [0.15-0.84] | 0.007 |
| Ongoing psychological care available vs only single sessions | 0.52 [0.05-0.98] | 0.66 [0.19-1.12] | 0.004 |
| Financing of the psychological care: fully covered vs patient needs to contribute to the costs | 0.60 [0.21-1.00] | 0.42 [0.02-0.82] | <0.001 |
| Psychological care at diagnosis vs no psychological consultation at T1D onset | 0.58 [0.30-0.85] | 0.73 [0.44-1.00] | 0.007 |

Data from linear regression models adjusted for confounders and with a random intercept for regions.

Regression analysis showed lower BMI SDS for PsyC centers with available ongoing psychological care compared to these where only single contact with a MHS is possible (0.51 [0.4-0.51] vs 0.68 [0.21-0.68], p=0.003), payment covered vs patients’ contribution to the consultation costs (0.61 [0.21-1] vs 0.38 [-0.03-0.78], p<0.001), use or not of a screening tool (0.49 [0.02-0.96] vs 0.56 [0.08-1.03], p=0.03), type of contact with the MHS at T1D diagnosis, and who can refer the patient to the MHS. All linear regression models on BMI SDS were implemented for aggregated data of each patient in 2019 and adjusted for age (categorized: <10 years, 10 to <14 years, ≥14 years), gender, age at type 1 diabetes onset (categorized: <6 years, 6 to <10 years, and ≥10 years), pump use (yes/no), number of SMBG (categorized: ≤4, >4, CGM), center size (categorized: ≤500 patients, >500 patients), HbA1c target (categorized: ≤7%, >7% or ≤53mmol/mol, >53mmol/mol) and completeness of documentation defined as data on ≥50% of patients available (yes/no). To take regional differences into account, a random intercept for regions with Cholesky variance structure and an optimization technique of Newton-Raphson with ridging was implemented. Regions were defined as: Europe, Asia & Middle East + Africa, North America, South America, Australia + New Zealand. To adjust for multiple comparisons the Tukey-Kramer method was used.
